# Digitally enabled acute care for atrial fibrillation: conception, feasibility and early outcomes of an AF virtual ward

**DOI:** 10.1136/openhrt-2023-002272

**Published:** 2023-06-29

**Authors:** Ahmed Kotb, Susanne Armstrong, Ivelin Koev, Ibrahim Antoun, Zakariyya Vali, Gaurav Panchal, Joseph Barker, Akash Mavilakandy, Shui Hao Chin, Merzaka Lazdam, Mokhtar Ibrahim, Alastair Sandilands, Riyaz Somani, G Andre Ng

**Affiliations:** 1 Department of Cardiovascular Sciences, University of Leicester, Leicester, UK; 2 Cardiology Department, University Hospitals of Leicester NHS Trust, Leicester, UK

**Keywords:** Atrial Fibrillation, Atrial Flutter, Arrhythmias, Cardiac

## Abstract

**Background:**

Atrial fibrillation (AF) represents a growing healthcare challenge, mainly driven by acute hospitalisations. Virtual wards could be the way forward to manage acute AF patients through remote monitoring, especially with the rise in global access to digital telecommunication and the growing acceptance of telemedicine post-COVID-19.

**Methods:**

An AF virtual ward was implemented as a proof-of-concept care model. Patients presenting acutely with AF or atrial flutter and rapid ventricular response to the hospital were onboarded to the virtual ward and managed at home through remote ECG-monitoring and ‘virtual’ ward rounds, after being given access to a single-lead ECG device, a blood pressure monitor and pulse oximeter with instructions to record daily ECGs, blood pressure, oxygen saturations and to complete an online AF symptom questionnaire. Data were uploaded to a digital platform for daily review by the clinical team. Primary outcomes included admission avoidance, readmission avoidance and patient satisfaction. Safety outcomes included unplanned discharge from the virtual ward, cardiovascular mortality and all-cause mortality.

**Results:**

There were 50 admissions to the virtual ward between January and August 2022. Twenty-four of them avoided initial hospital admission as patients were directly enrolled to the virtual ward from outpatient settings. A further 25 readmissions were appropriately prevented during virtual surveillance. Patient satisfaction questionnaires yielded 100% positive responses among participants. There were three unplanned discharges from the virtual ward requiring hospitalisation. Mean heart rate on admission to the virtual ward and discharge was 122±26 and 82±27 bpm respectively. A rhythm control strategy was pursued in 82% (n=41) and 20% (n=10) required 3 or more remote pharmacological interventions.

**Conclusion:**

This is a first real-world experience of an AF virtual ward that heralds a potential means for reducing AF hospitalisations and the associated financial burden, without compromising on patients’ care or safety.

**WHAT IS ALREADY KNOWN ON THIS TOPIC**

- Virtual wards have gained significant attention during the COVID-19 pandemic. Remote monitoring is an established practice for arrhythmia detection and outpatient follow-up. Yet, there is a real paucity of data when it comes to hospital in-patients with atrial fibrillation (AF) and whether they can receive equivalent levels of care remotely.

**WHAT THIS STUDY ADDS**

- We implemented a virtual ward for AF patients either through promoting early discharge or as an alternative to hospital admission. We provided a reproducible virtual healthcare model for AF patients, which was effective, safe and received positive feedback from patients.

**HOW THIS STUDY MIGHT AFFECT RESEARCH, PRACTICE OR POLICY**

- This novel model of healthcare delivery may provide an alternative to the traditional in-patient care for patients with AF, thereby reducing the huge associated economic burden on healthcare without compromising patients’ care. Work is still needed to further confirm longitudinal safety and cost-effectiveness, to facilitate creating a future blueprint for digitally enabled acute AF virtual wards.

## Introduction

Atrial fibrillation (AF) is the most common cardiac arrhythmia associated with a significant health and economic burden.^1^ England’s Quality and Outcomes Framework 2020–2021 report published in September 2021 estimates a population of at least 1.2 million patients in England that were on treatment for AF, (indicating a prevalence of 2%).^2^

Burdett and Lip predicted estimated direct costs of AF, as a percentage of the UK’s National Health Services (NHS) expenditure, at between £1.435bn (0.91%) to £2.548bn (1.62%) in the year 2020, increasing to £2.351bn (1.1%) to £5.562bn (2.63%) for 2030 and £3.851bn (1.35%) to £12.143bn (4.27%) in 2040. The majority of these direct AF costs (nearly 60%) are driven by inpatient hospital admissions.^3^

Under standard care, patients presenting to emergency departments (ED) with AF or atrial flutter (AFL) are often admitted to hospital to achieve control of their arrhythmia. The observation period might range from hours to days despite patients often being asymptomatic or only mildly symptomatic and haemodynamically stable.

Based on locally derived financial calculations, the cost to the local health ecosystem for a patient to stay in a monitored hospital bed for one night, is estimated at £630. Furthermore, patients with AF are often admitted across multiple medical specialties and may not always receive the required specialist care, resulting in a convoluted patient journey and delays in receiving definitive management plans.

The deficiencies in the current model of care, together with the financial implications outlined above, highlighted the importance of exploring the use of innovative systems to combat the emerging AF pandemic. The advent of technology and its growing sociocultural acceptance in a postpandemic world has facilitated the introduction of remote arrhythmia management in healthcare.

The mobile AF application II trial investigated the value of a holistic care approach in AF patients, including the AF Better Care pathway (ABC pathway), by combining mobile smart technologies and the Huawei smartwatch, and demonstrated lower adverse events in the form of a composite outcome of stroke, thromboembolism, all-cause death and rehospitalisation (HR, 0.37; 95% CI 0.26 to 0.53; p<0.001), among AF patients with multimorbidity.^4^ The aim of this work was to take this one step further, by implementing a virtual ward that delivered the same holistic approach to care for patients with AF, through the use of multidisciplinary teams and remote technologies, and to assess the clinical utility, safety and financial savings that may be derived from such a model.

## Methods

### Study design

An ambulatory virtual ward was implemented at Glenfield Hospital, a UK tertiary cardiorespiratory centre, as a proof-of-concept model of care following local safety and governance approvals with the hospital institutional board and after completing a data protection impact assessment.

We prospectively identified consecutive AF cases eligible for virtual ward enrolment that presented to the hospital between 31 January 2022 and 25 August 2022.

### Context

The current clinical pathway of care at our centre required patients who present to ED with AF or AFL and fast ventricular response to be admitted to a hospital ward for monitoring. Patients might also self-present or are referred by their primary care provider directly to our acute cardiorespiratory unit where they stay in hospital until rate or rhythm control is achieved.

Locally, 1333 patients with a primary diagnosis of AF were admitted to the University Hospitals of Leicester NHS Trust (UHL), a UK tertiary centre, between March 2019 and February 2020, with a 10% annual increase. An initial retrospective analysis was carried out on all primary AF-related admissions to our hospital’s acute cardiorespiratory admissions unit, between 1 August 2021 and 30 September 2021. This analysis aimed to screen AF-related admissions to identify a cohort that were haemodynamically stable and demonstrated an absence of other indicators for hospitalisation. Two-hundred and eleven patients were identified, averaging a length of stay of between 2 and 5 days, of which 50% (n=105) were potential candidates for an AF virtual ward.

### Study population

Admission criteria to the virtual ward were as follows: patients above the age of 18 with a primary diagnosis of AF or flutter (AF/AFL being the only acute clinical issue) with a duration of >48 hours at the time of referral, or unknown, no underlying acute factors causing AF (ie, heart failure/thyroid dysfunction/sepsis), haemodynamically stable with no other clinical indicators for hospital admission (eg, acute coronary syndrome, pulmonary embolism), heart rate (HR) less than or equal to 140 bpm, able to read and speak English or receive support from a relative, resident in the UK and have an active telephone number (online supplemental table 1). Referrals were received from ED, acute cardiorespiratory units, outpatient department, cardiac investigations department and hospital wards. Virtual ward admissions were either ‘step-up’, where patients were directly onboarded to the virtual ward instead of hospitalisation, or ‘step-across’ to facilitate early hospital discharge for AF in-patients by continuing their acute care on the virtual ward.

10.1136/openhrt-2023-002272.supp1 Supplementary data

### Digital platform and devices for the virtual ward

**Figure 1:**
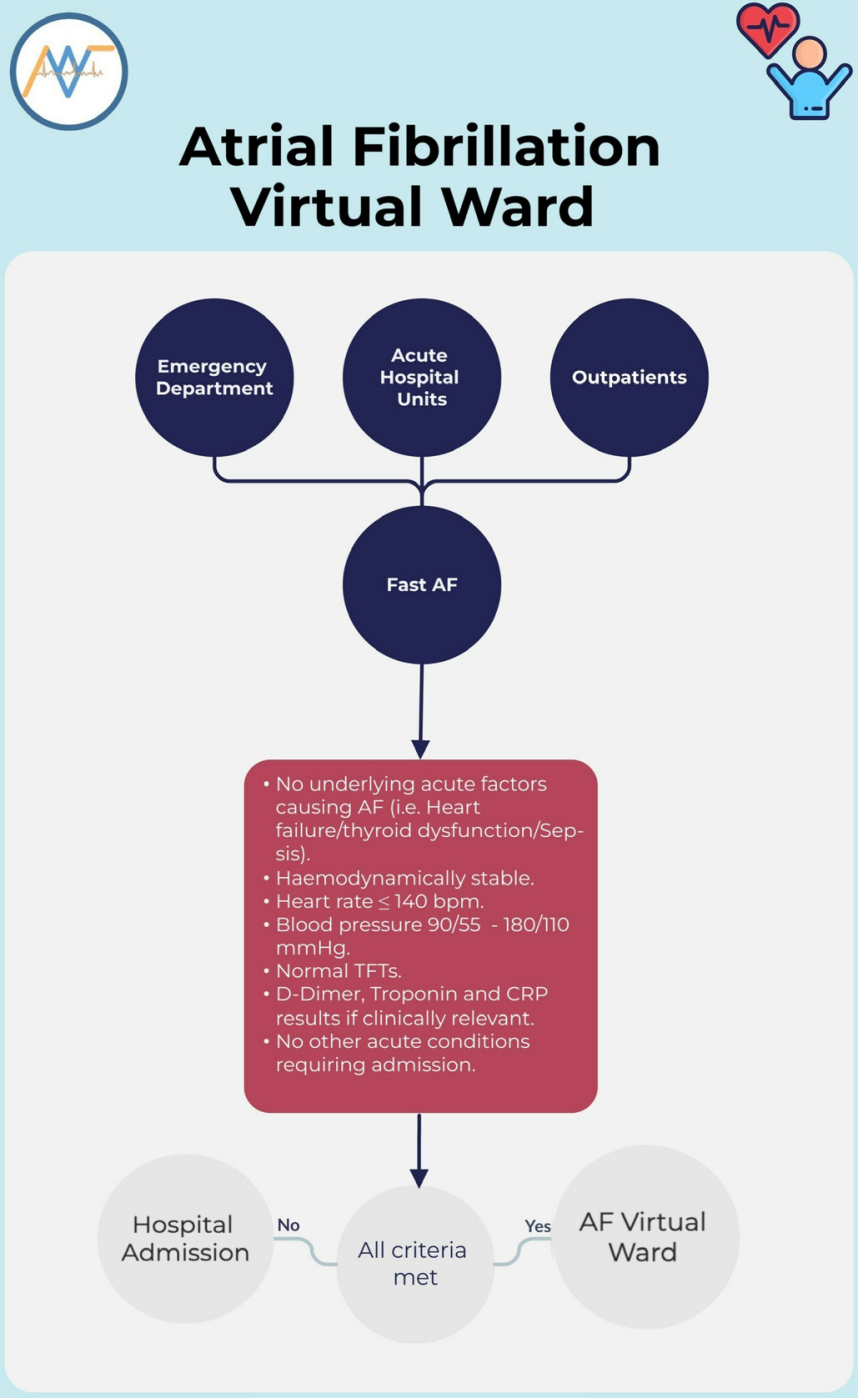
Admission criteria to the virtual ward. AF, atrial fibrillation; CRP, C reactive protein; TFT, thyroid function tests.

Once identified as suitable for onboarding onto the virtual ward (figure 1), patients were given access to a single lead Kardia ECG device (AliveCor, Mountain View, California, USA), a Bluetooth-integrated blood pressure machine (Fora P30, ForaCare Suisse AG) and pulse oximeter (ChoiceMMed, Beijing). The Kardia ECG device has been approved by the National Institute for Health and Care Excellence (NICE) for AF detection and has been previously validated against three other portable devices, showing an overall accuracy of 96.65% (95% CI 94.44% to 98.16%).^5^

**Figure 2:**
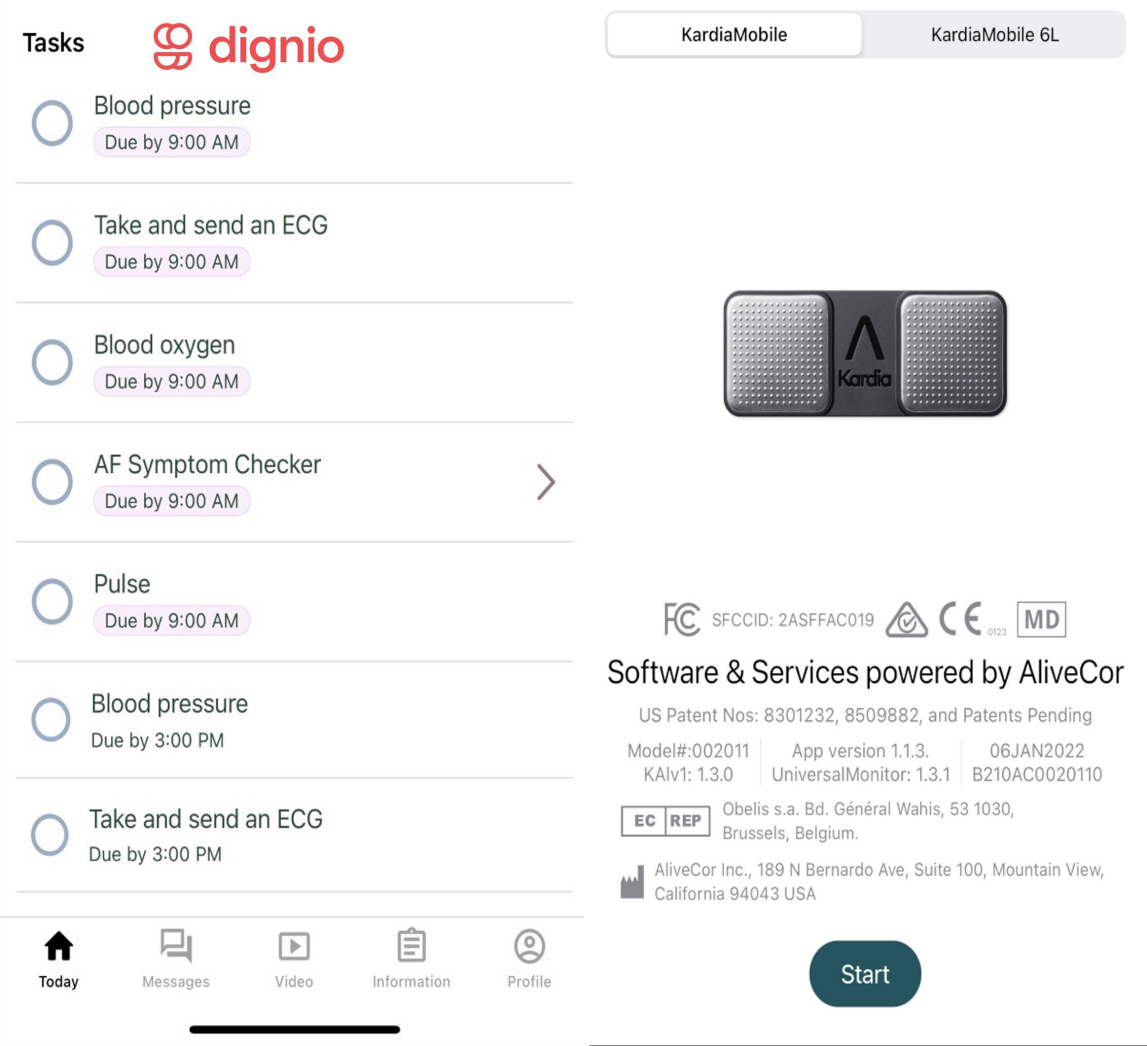
Patient’s interface. AF, atrial fibrillation.

**Figure 3:**
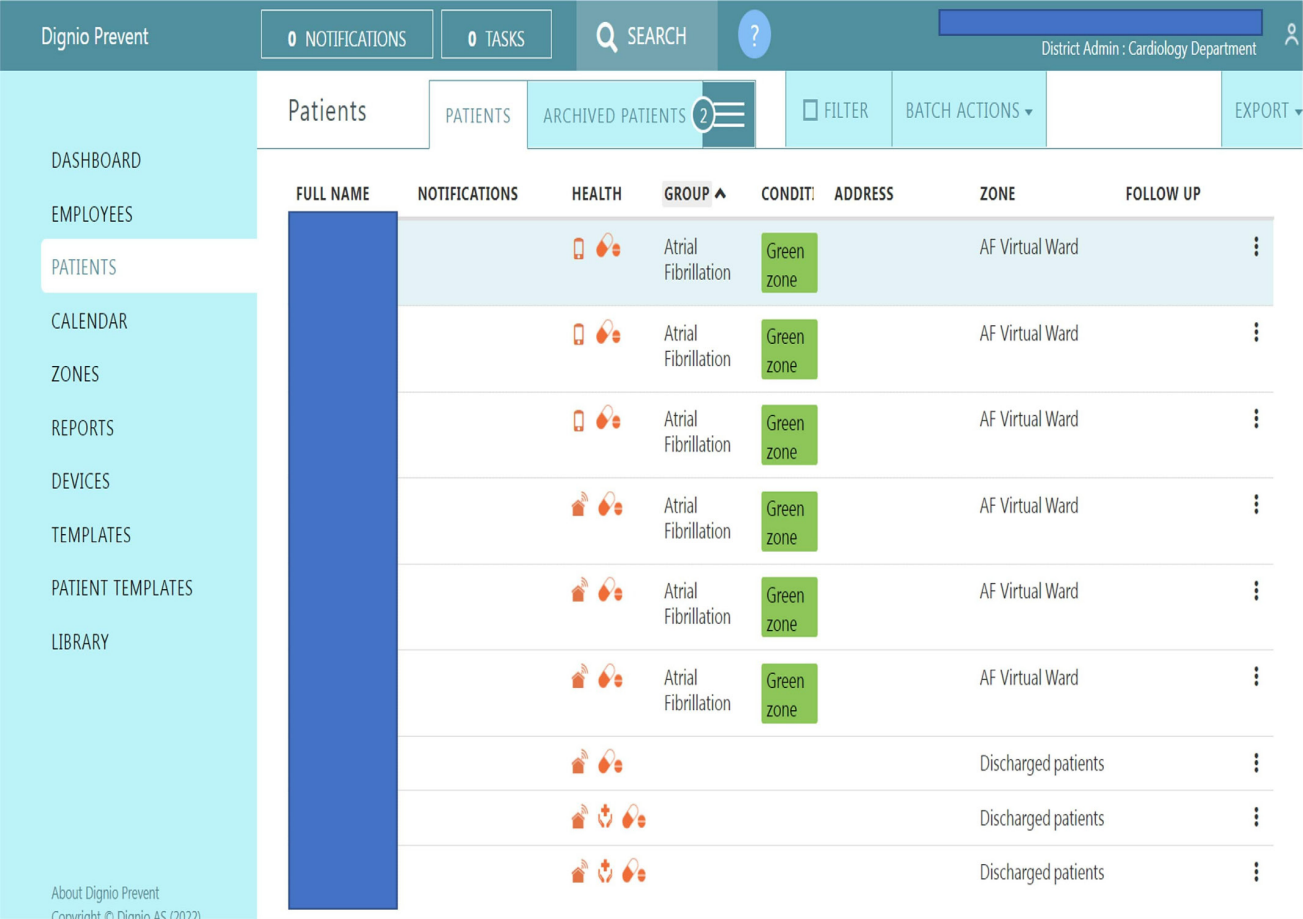
Online platform (physician interface). AF, atrial fibrillation.

All patients received a set of tasks to complete each day via a smartphone app (MyDignio by Dignio, UK) accessible by download (figure 2). They were instructed to record their ECG, blood pressure, oxygen saturation and fill an online AF symptom questionnaire. Clinical data were uploaded through the app to an online platform (Dignio Prevent, Dignio) for review by the clinical team (figure 3). ECG readings were separately captured by the Kardia single-lead ECG and automatically uploaded to a separate ECG digital platform (KardiaPro), provided by AliveCor (Mountain View). Electronic tablets were provided to patients if needed, to enable maximum inclusivity.

Patients received a reminder 15 min before each task was due to be completed. If the task was not completed, a notification was sent to the online platform dashboard, which prompted the specialist team to ask the patient to complete the task.

### Clinical oversight

The clinical team consisted of two arrhythmia advanced care practitioners, three cardiology registrars and six consultant cardiologists who subspecialise in cardiac electrophysiology.

A treatment plan was formulated at the onboarding stage and patients were sorted into green, amber or red zones according to the required intensity of monitoring, which was determined by the practitioner’s clinical judgement. Green-zone patients required submission of twice-daily readings, while red and amber patients were asked to provide measurements three times per day. Red zone patients required an additional daily video consultation until they were de-escalated to a lower zone. In addition, the traffic light system was used in categorising patients’ observations with certain thresholds set for blood pressure, oxygen saturation and HR. Threshold breaches were automatically flagged on the platform as amber or red notifications depending on their classification as minor or major breaches of thresholds.

‘Virtual ward rounds’ were carried out daily by the clinical team with patients reviewed 7 days per week, 09:00 – 17:00 hours and communication was conducted through asynchronous in-app messaging, synchronous telephone or video consultations. Medication adjustment was arranged through the hospital pharmacy with an option of home delivery if required to avoid repeated hospital visits.

### Out-of-hours clinical care

All patients were given access to a self-management plan displayed through the app which included instructions to follow in case of any out-of-hours (OOH) urgent clinical queries. Where appropriate, a pill-in-pocket approach was adopted for OOH breakthrough symptoms. Patients were given the option to contact the on-call cardiology registrar if urgent advice was needed, in addition to a list of red-flag symptoms that would warrant calling the emergency services.

### Discharge from the virtual ward

**Figure 4:**
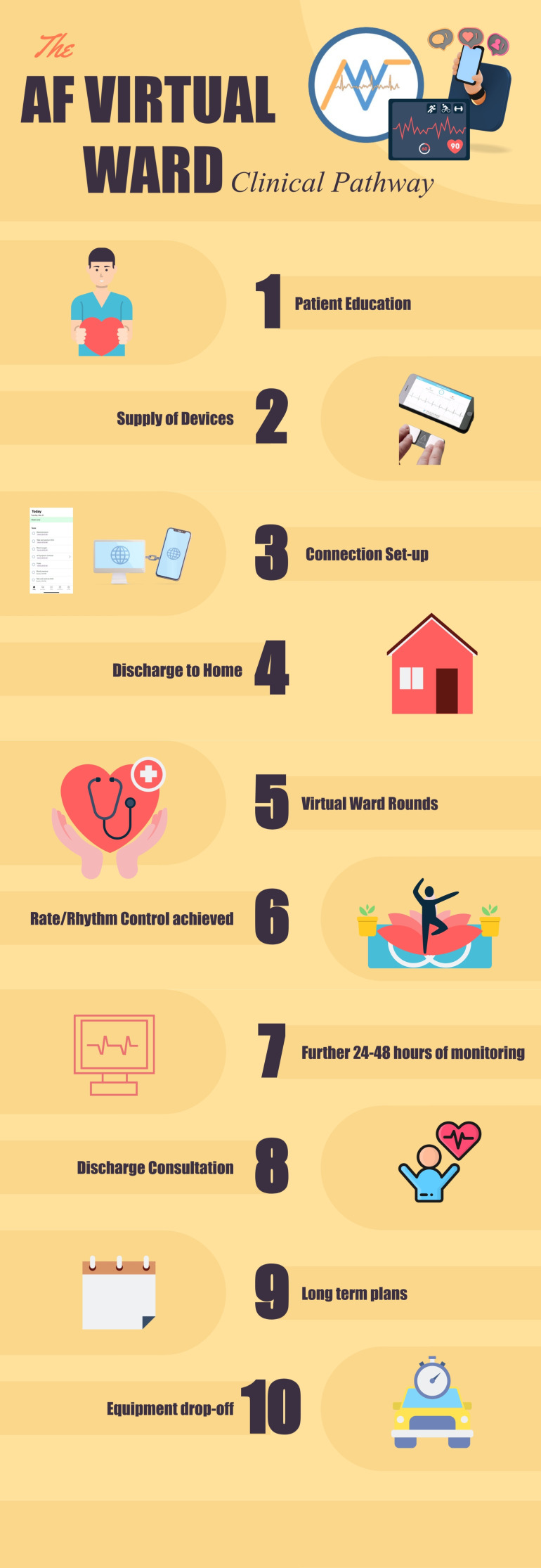
AF virtual ward clinical pathway. AF, atrial fibrillation.

Discharge was classified into planned discharge for patients who were satisfactorily treated and safely discharged home, or unplanned discharge for those who decided to unilaterally terminate the monitoring because of service dissatisfaction or those who required hospital readmission for clinical reasons. A face-to-face discharge consultation was arranged at the end of monitoring for equipment return and long-term plans discussion. Pathway Summary is demonstrated in figure 4.

### Outcomes

Anonymised data were collected for all patients admitted to the AF virtual ward between 31 January 2022 and 25 August 2022. Observational outcomes were collected to assess the efficacy and safety of the virtual ward. Primary outcomes included admission avoidance, readmission avoidance using the index admission clinical criteria as the parameter for readmission likelihood and patient satisfaction. Secondary outcomes included length of stay, AF management strategies (rate vs rhythm control) and referrals for elective procedures. Safety outcomes included unplanned discharge from the virtual ward, cardiovascular mortality and all-cause mortality.

Patients’ satisfaction was assessed using the NHS friends and family test (FFT), where patients were asked about their experience of the service they received and how likely they were to recommend it to friends and family if they needed similar care or treatment. The questionnaire employed a Likert-type scale with input options including very good, good, neither good nor poor, poor, very poor and don’t know. The responses to the FFT question were used to create an overall score of service satisfaction. Patients were also allowed to provide narrative free-text feedback.

### Statistical analysis

Data were stored using Microsoft Excel 2013, while statistical analysis was conducted using GraphPad Prism V.9.3 (San Diego, California, USA). Categorical variables were expressed as frequency and percentage. Median and IQR were used to describe non-parametric data while mean and SD was used to describe parametric data. The normality of distribution was decided by the D’Agostino-Pearson normality test. Unpaired t-tests or Mann-Whitney U test were utilised to analyse unmatched data according to the normality of distribution. Patients’ parameters changes and outcomes were analysed using Wilcoxon matched-pairs signed-ranks test or paired t-test depending on the data’s distribution. A p<0.05 was considered statistically significant.

## Results

Forty-seven patients were deemed eligible for enrolment into the virtual ward between 31 January 2022 and 25 August 2022. One patient declined admission because of technology-related anxiety. Two patients were admitted twice to the virtual ward and one patient was admitted three times, generating 50 admissions that were included in the analysis. All patients admitted to the virtual ward would otherwise have been admitted to hospital (according to the current standard of care) or were in-patients receiving treatment on cardiology wards.

Most admissions were males, 68% (n=34). Two patients were from an ethnic minority group (Asian-British). The mean age on admission was 66±9.5 years, 18% of patients (n=9) were diabetic, 42% (n=21) were hypertensive, 4% (n=2) had been diagnosed with obstructive sleep apnoea and 10% (n=5) of patients had a thyroid-disorder requiring treatment (table 1). Three patients had disabilities, two patients were registered blind and one patient was a wheel-chair user. The mean length of stay in the virtual ward was 20±13 days. Demographics on admission and discharge from the virtual ward are presented in tables 1 and 2.

**Table 1:** Demographics on admission to the virtual ward

Demographic	Value
Males	34 (68%)
Age (years)	66±9.5
AF on admission	39 (78%)
AFL on admission	9 (18%)
Diabetes mellitus	9 (18%)
Hypertension	21 (42%)
Obstructive sleep apnoea	2 (4%)
Ischaemic heart disease	5 (10%)
Thyroid dysfunction	5 (10%)
On-boarding BP (mmHg)	(136±20)/(95±16)
On-boarding HR (bpm)	122±26

AF, atrial fibrillation; AFL, atrial flutter; BP, blood pressure; HR, heart rate.

**Table 2:** Demographics on discharge from the virtual ward

Demographic	Value
AF on discharge	25 (50%)
AFL on discharge	6 (12%)
SR on discharge	19 (38%)
Off-boarding BP (mmHg)	(121±16)/(82±9.8)
Off-boarding HR (bpm)	88±27
Rhythm control strategy	41 (82%)
Started on AAD	24 (48%)
DCCV referral	14 (28%)
Ablation referral	15 (30%)
FFT participation	45 (90%)
FFT satisfaction	100%
Mean length of stay (days)	20±13

AAD, antiarrhythmic drug; AF, atrial fibrillation; AFL, atrial flutter; BP, blood pressure; DCCV, direct current cardioversion; FFT, friends and family test; HR, heart rate; SR, sinus rhythm.

Forty-eight per cent (n=24) of admissions were taken directly from outpatient sources (ECG clinic, Pacemaker clinic, Echocardiography suite, preprocedure assessment clinics and outpatient clinics) hence initial hospital admissions were completely avoided, and 25 readmissions were avoided, preventing a total of 49 hospitalisations and saving 98–245 hospital bed days. Mean HR at the time of admission and discharge was 122±26 bpm and 88±27 bpm, respectively. The majority of patients on admission exhibited AF (78%, n=39) or AFL (18%, n=9), with two patients in sinus rhythm, yet with frequently recurring bouts of AF (4%, n=2). A rhythm control strategy was pursued in 82% (n=41, p<0.0001), with 48% (n=24, p=0.84) being started on antiarrhythmic drugs and 20% (n=10) required 3 or more remote pharmacological interventions, defined as any instance where medications were altered, or the dose titrated to improve patients’ symptoms and/or optimise rate control.

Thirty-eight per cent of admissions were in sinus rhythm on discharge (n=19). Over a quarter of patients, 28% (n=14) were referred for direct current cardioversion and 30% (n=15) were referred for catheter ablation. There were three unplanned discharges (6%) which were all due to hospital readmission. Reasons for readmission were tachycardia requiring acute cardioversion, deteriorating renal function requiring hospitalisation in a patient with pre-existing chronic kidney disease, and significant fluid overload requiring intravenous diuresis. Only one patient contacted the on-call cardiology registrar OOH for urgent advice related to slow HR, and no hospital admission was required. There was one sudden unexpected death, not related to their care. A formal autopsy was not performed, and the cause of death was attributed to hyperkalaemia. There was no clinical deterioration immediately prior to the unexpected death with the last virtual ward ECG recording sinus rhythm at 74 bpm, a stable haemodynamic and symptom profile. This patient was not on any nephrotoxic medications with normal levels of serum electrolytes on hospital discharge and no clinical indication for repeat serum potassium level assessment.

Patients required prompting at least once to complete a task in 10 (20%) virtual ward admissions; 6 patients required prompting once, 2 patients required prompting twice and only 1 patient required prompting three times.

**Figure 5:**
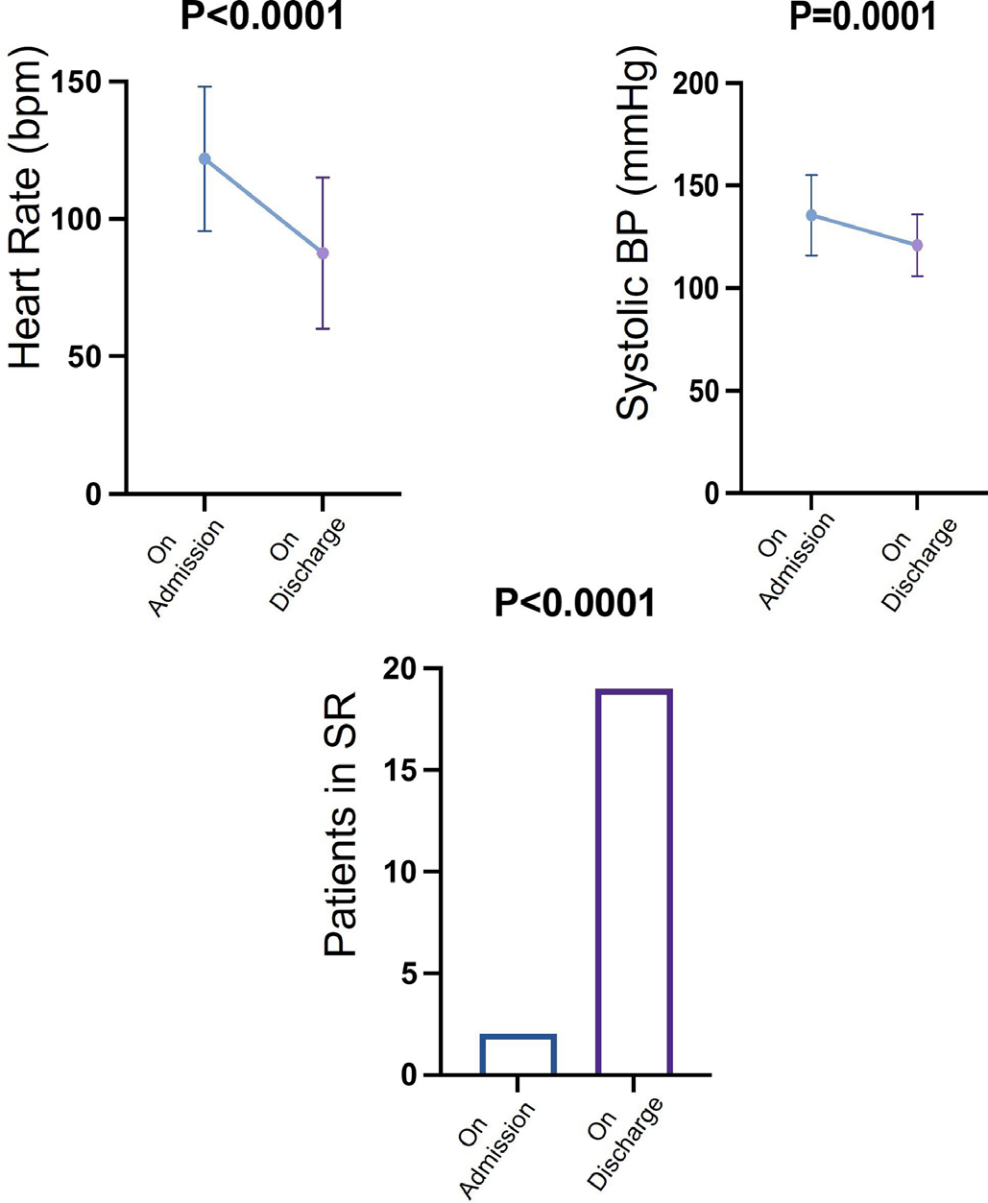
Clinical parameters on admission and discharge from the virtual ward. SR, sinus rhythm

The FFT participation was 90% (n=45) yielding 100% positive responses with all FFT participants recommending the service. Examples of narrative feedback can be found in online supplemental table 2. The main themes of positive feedback included being able to avoid staying in hospital, feeling empowered, active participation in care and easy accessibility to healthcare staff. Negative comments were mainly focused on the time taken to be discharged from in-patient hospital services after being onboarded to the virtual ward, initial technical difficulty with the equipment before becoming familiar, and a request for more clarity in text messages. A summary of the results is illustrated in figure 5, tables 1 and 2.

## Discussion

In this study, we present the first real-world experience of a virtual ward for hospital patients exhibiting AF with a fast ventricular response. It illustrates that the use of digital technology can support the provision of efficient clinical care outside of the traditional bricks-and-mortar hospital setting for this population. The aim of this virtual ward was not only to provide comparable levels of safety and efficacy to hospital-based care, acting as an alternative to hospital admission, but also to facilitate relatively longer (virtual) stays, creating newer models where the boundaries between community and hospital-led care were merged, allowing for a unified patient-centred care approach. Patients on the virtual ward were treated as they would be in a hospital bed, in accordance with the European Society of Cardiology 2020 AF guidelines,^6^ and the NICE 2021 guidelines for AF diagnosis and management.^7^

Out of 50 virtual admissions, 24 were identified and on-boarded to the virtual ward instead of the usual pathway of hospital admission, while the remaining 26 admissions comprised of in-patients discharged earlier from the hospital setting with plans for prolonged remote monitoring through the virtual ward. This included two patients who were frequently relapsing between AF and sinus rhythm while in hospital and were at risk of imminent re-admission, following hospital discharge.

It was challenging to assess how many rehospitalisations were avoided per every virtual admission. Any instance of initial clinical presentation recurrence (ie, the initial HR, symptoms and blood pressure that resulted in the patient’s index admission to hospital) where hospital admission was deferred because of a pharmacological intervention implemented virtually by the clinical team, was counted as a readmission avoidance. A total of 25 rehospitalisations were, therefore, prevented, according to this definition.

In total, these 50 virtual admissions resulted in 49 actual hospital admissions being avoided. With a usual length stay of 2–5 days for AF patients in a hospital bed in the routine care pathway, a total of between 98 and 245 bed-days were saved. This may justify the relatively prolonged length of stay per patient in this virtual ward, which can be further explained by two key considerations. First, this was a pilot study with a learning curve and a new process that required adjustment time for patients and staff to gain confidence. Second, the prolonged admissions allowed provision of a more integrated holistic approach to care and the formulation of long-term personalised plans. Nonetheless, the length of stay is expected to reduce as the service matures.

Furthermore, this novel pathway and the utilised applications were well received by patients, with the main negative comments addressing the initial difficulty with technology and expressing the need for more instant communication. The need for more communication might have been aggravated by the lack of continuous visibility of healthcare staff compared with the standard of care in hospital. Another theme to emerge were negative comments related to the physical discharge processes after being onboarded to the virtual ward, and while we acknowledge that this was not directly under our control, we believe that there is scope for improvement once the new pathway is well integrated. Despite these remarks, there was obvious endorsement from patients regarding the service, with none of the patients asking to stop the monitoring prematurely and all FFT participants saying they would recommend the service. Additionally, ensuring digital equity was a key factor during the set-up of this virtual ward as patients who did not possess smartphones were provided with electronic tablets on loan to use for sending measurements following demonstration on using the equipment. Digital inclusion was further demonstrated with enrolling patients in their 80s, along with two patients being registered blind.

Difficulties in dealing with technology remains one of the major obstacles facing the establishment of remote care pathways. Weng *et al* demonstrated a promising impact of virtual pathways on ambulatory care for AF patients referred for specialist care because of new-onset and/or symptomatic AF, yet the primary difficulty faced was patients not being able to use computer technology.^8^ In our study population, one patient declined to go on the virtual ward because of technological-related anxiety. This number is likely to increase with the wider adoption of the service and further measures to familiarise patients with the technology may be needed.

The service was secondary care-focused, where patients receive specialist-led care and management plans. This was reflected in a rhythm control strategy being adopted in the majority of patients with more than one-third of the patients being discharged in sinus rhythm due to spontaneous reversion. This is in line with recent evidence suggesting the favourable impact of early rhythm control on adverse cardiovascular events.^9^ Moreover, patients were discharged with definitive management plans and early destination therapy decisions with a third of the admissions culminating in a cardioversion referral and a third referred for catheter ablation.

Of note, remote management did not prevent medication optimisation or uptitration as one in five admissions received at least three remote pharmacological interventions.

When it comes to safety, the presence of three unplanned discharges resulting in readmissions, potentially illustrates a real-world practice and provides reassurance to healthcare professionals and patients that vigilance towards safety is among the hallmarks of this pathway as prompt escalation of care would be implemented if indicated.

There have been earlier trials at the establishment of virtual pathways for ambulatory care of AF, which mainly focused on outpatients referred for specialist care because of AF,^8^ or post-AF ablation virtual follow-up clinics.^10^ This study, however, presents the first AF virtual ward dealing with hospital-level patients with AF. While we acknowledge that more work is still needed, it provides a promising telemedicine-based care model that is able to provide safe and efficient patient-centred clinical care.

### Limitations

Our study has several limitations. First, our population was predominantly Caucasian with only two patients of an ethnic minority group (Asian-British). This could be explained by the higher prevalence of AF among white ethnicity compared with other ethnic groups.^11^ This was an observational study with a lack of randomisation. It had a small sample size which was not derived from a formal power calculation, owing to inclusion of all patients meeting the study criteria, with the absence of a matched control group. Our analysis was focused on immediate outcomes and thus as a result, deficient in longitudinal follow-up data. Furthermore, the small sample size and the variable length of stay did not allow an accurate evaluation into cost-effectiveness and economic analysis. However, this was aimed to be a proof-of-concept study. Further work is still ongoing in a larger patient cohort with a matched control arm.

## Conclusions

This proof of concept is the first real-world experience of a virtual ward for AF. It provides a promising new telemedicine-based work model for patients with fast AF, as an alternative to traditional in-patient care and with a clear appetite among both patients and health professionals. It demonstrates a potential to reduce the financial and backlog pressures caused by AF admissions without compromising patients’ care or safety. Work is ongoing to further confirm the longitudinal safety and cost-effectiveness in the context of a larger patient cohort. This may support development of virtual wards into future models of healthcare delivery that can morph and adapt according to different organisational workflows in various localities and healthcare systems, to consequently be replicated across the UK and beyond—not only for AF, but also for other chronic diseases that continue to impose a significant burden on healthcare.

## Data Availability

Data are available on reasonable request. The study team would be able to share AF virtual ward start dates and anonymised admission data with the agreement of University Hospitals of Leicester information governance and privacy department. Sources for data that are already publicly available are supplied either in the text or the references.
