# Supplementary material for: Digitally enabled acute care for atrial fibrillation: conception, feasibility and early outcomes of an AF virtual ward
Source: Open Heart. 2023 Jun 29;10(1):e002272. doi: 10.1136/openhrt-2023-002272 (PMC10314691; doi:10.1136/openhrt-2023-002272)
Supplement: Supplementary data [file openhrt-2023-002272supp001.pdf]

Table 1 Admission criteria to the virtual ward.

**Inclusion criteria**	**Exclusion criteria**
Patients above the age of 18 presenting with AF or AFL as the main indication for admission.	Patients who are adequately rate controlled with a HR <100 bpm on referral.
No underlying acute factors causing AF (i.e. Heart failure/thyroid dysfunction/Sepsis).	Patients who revert to sinus rhythm before on-boarding to the virtual ward.
AF/AFL duration > 48 hours at the time of referral or unknown	Patients on the waiting list for an in-patient procedure that prevents hospital discharge.
Haemodynamically stable with no other clinical indications for hospital admission. e.g. acute coronary syndrome, pulmonary embolism.	
Heart rate less than or equal 140 bpm.	
Blood pressure more than or equal 90/55 and less than 180/110mmHg.	

Completion of baseline observations, Thyroid Function Test (result may be pending) and as clinically relevant CRP, D-Dimer, Troponin with results being clinically insignificant.	
Patient willing and agreed to engage with virtual monitoring.	
Patient willing and agreed to return all loaned equipment.	
Able to manage technology or receive support from a relative.	
Able to read and speak English or receive support from a relative.	
Patient remains in the United Kingdom.	
Patient must have an active telephone number.	

AF *Atrial Fibrillation*; AFL *Atrial flutter;* CRP *C-Reactive protein*

Table 2. Summary of feedback from patients on discharge from the virtual ward, positive and negative with examples.

**Positive**	
Experience with a virtual ward	It gave me the feeling of confidence that someone was monitoring me whilst my condition stabilised. There were two or three times where I felt that would have attended ED or rang emergency services if it wasn't for the support that I have received.
	Must say though, that this is much better than being in and taking up a bed and your time, far better.
	It made me feel I was still being cared for although I was not in hospital. I was very concerned when I was admitted, and this made my whole experience very good.
Patient engagement and education	It gives us a chance to be more involved in treatment and understanding the problem and allowing us some control which I often think is missing.
	It provided good support and gave me information about my condition.
Technology easy to use	I found it very easy to submit the readings.
	I am technophobe and I found it easy.

	Equipment was simple to use, and the feedback was helpful and reassuring.
Arranging re-admission to hospital	We and the paramedic who had to visit were really impressed with the service.
**Negative**	
Difficulties with the technology	It was a bit confusing at first but once you are comfortable and familiar with the technology it gives you confidence in your recovery.
	Explanation was very good but possibly a bit rushed.
	I feel like elderly patients might get confused with the technology.
	I found it difficult at first but was able to get support from staff once I got home.
Discharge from inpatient services	Felt very stressed waiting for 23 hours for the hospital paperwork to be completed so that I can go home.
	The hospital ward was disorienting, and I had no idea where I was.
Communication	Make sure text messages sent are clear in what they mean.
	Make the contact number clearer in case you need urgent help, messages seem to take some time.

Suggestions for improvement	Do several tests on the equipment and familiarise myself with the equipment before being discharged.
	The timings for readings were restrictive, it would be better if they were first thing in the morning, after lunch and bedtime if possible.
